# Intraoperative intravitreal triamcinolone acetonide injection for prevention of postoperative inflammation and complications after phacoemulsification in patients with uveitic cataract

**DOI:** 10.1186/s12886-021-02017-y

**Published:** 2021-06-04

**Authors:** Yan Ren, Shufang Du, Dongping Zheng, Yanyun Shi, Luping Pan, Hua Yan

**Affiliations:** 1 Department of Ophthalmology, Tianjin Medical University General Hospital, No. 154, Anshan Road, Tianjin, 300052 China; 2 Department of Fundus Diseases, Shanxi Eye Hospital, Taiyuan, 030002 Shanxi China

**Keywords:** Triamcinolone acetonide, Intravitreal injection, Inflammation, Uveitic cataract, Phacoemulsification

## Abstract

**Background:**

We aimed to evaluate the efficacy and safety of phacoemulsification with intravitreal 3 mg triamcinolone acetonide injection in preventing postoperative inflammation and complications in patients with non-infectious anterior uveitis and panuveitis complicated cataract.

**Method:**

In this retrospective cohort study, 140 uveitic cataract patients who received phacoemulsification and intraocular lens implantation in Shanxi Eye hospital from January 2018 to January 2020 were reviewed. The IVTA group (51 eyes of 41 patients) received intravitreal injection of 3 mg triamcinolone acetonide (TA) at the end of surgery, and the control group (51 eyes of 41 patients) without injection matched by propensity score matching were enrolled. Outcome measures were best corrected visual acuity (BCVA), anterior chamber inflammation, intraocular pressure, corneal endothelial cell density, central macular thickness and complications within 3 months follow-up.

**Results:**

The degree of postoperative anterior chamber inflammation in the IVTA group was lighter than that in the control group (*P* < 0.05). The postoperative logMAR BCVA of anterior uveitis was better and improved more quickly in the IVTA group(*P* < 0.05). Postoperative time of using corticosteroids was shorter in the IVTA group as compared to the control group (*P* < 0.05). The central macular thickness at postoperative month 1 was statistically significantly lower in the IVTA group (*P* < 0.05). There were no statistically significant differences between the two groups in postoperative corneal endothelial cell density and intraocular pressure (*P* > 0.05). Two of 51 eyes (3.9%) in the IVTA group and 8 of 51 eyes (15.7%) in the control group had recurrence of uveitis; 6 of 45 eyes (13.3%) in the control group developed cystoid macular edema but none in the IVTA group; 11 of 51 eyes (21.6%) in the IVTA group and 22 of 51 eyes (43.1%) in the control group developed posterior synechiae postoperatively.

**Conclusions:**

Intraoperative Intravitreal injection of 3 mg TA is an effective and safe adjunctive therapy for preventing postoperative inflammation and complications to promote early recovery for anterior uveitis or panuveitis complicated cataract patients following phacoemulsification.

**Trial registration:**

This retrospective cohort study was in accordance with the tenets of the Helsinki Declaration and was approved by the Shanxi Eye Hospital Ethics Committee. Written informed consent was obtained from all participants for their clinical records to be used in this study.

## Background

Uveitis is a leading cause of blindness worldwide, and the development of cataracts is a high-incidence complication due to the intraocular inflammation and/or consequence of long-term treatment with topical, regional and systemic corticosteroids [1–3]. Phacoemulsification with implantation of intraocular lens may be effective in visual rehabilitation but performing cataract surgery in eyes with uveitis has historically been a challenge, being technically more difficult and providing less reliable outcomes than senile cataract. The visual outcome of cataract surgery can be unpredictable due to a greater incidence of recurrent inflammation and postoperative complications, such as macular edema, posterior capsule opacification (PCO) and glaucoma [4, 5].

Preoperative and postoperative use of topical and/or systemic corticosteroids is general treatment when patients with non-infectious uveitis receive cataract surgery to restrain the postoperative inflammation. Systemic corticosteroids are effective in the treatment of posterior uveitis, bilateral uveitis and chronic uveitis associated with an underlying systemic disease. However, systemic corticosteroids therapy may accompany significant side effects including gastrointestinal ulcers, osteoporosis, hyperglycaemia, Cushing syndrome, infections and electrolyte disturbance [6].

Intravitreal administration of TA offers the maximal drug efficacy and the lowest risk of systemic side effects. Successful application of intravitreal TA injection has been confirmed in treating patients with inflammation and macular edema associated with posterior uveitis [7, 8]. Results of some case reports also showed optimistic outcomes after intravitreal TA injection combined with cataract extraction in anterior uveitis patients [5, 9]. But the the risk of high intraocular pressure was still inevitable and dose-dependent, and potential toxicity such as to the corneal endothelium is also concerned after phacoemulsification. Whether low dose TA could be effective and reduce the risk of side effects has not been reported in patients with panuveitis and anterior uveitis undergoing phacoemulsification.

To our knowledge, there are limited data available in the literature about the preventive effectiveness and adverse effects of 3 mg TA intravitreal injection for the treatment of uveitic cataracts. In this retrospective study, we have compared the postoperative outcomes of phacoemulsification with and without intravitreal injection of 3 mg TA in treatment of cataract patients suffering from chronic anterior uveitis or panuveitis in the real world. This is the first of its kind, retrospective cohort study in which propensity score matching (PSM) have been used to adjust potential confounding factors and screened suitable cases to establish the efficacy and safety of intravitreal TA injection combined with phacoemulsification in preventing the postoperative inflammation and complications in uveitits cases.

## Patients and methods

This retrospective cohort study was in accordance with the tenets of the Helsinki Declaration and was approved by the Shanxi Eye Hospital Ethics Committee. Written informed consent was obtained from all participants for their clinical records to be used in this study, including demographic characteristics, clinical manifestations and treatment procedures.

Patients with non-infectious uveitis complicated cataract who were subjected to phacoemulsification and primary posterior capsule implantation of intraocular lens (IOL) in Shanxi Eye Hospital from January 2018 to January 2020 were enrolled in this study. The IVTA group received intravitreal injection of triamcinolone acetonide (3 mg/0.075 mL) intraoperatively, and the control group didn’t.

Inclusion criteria were: (i) the diagnosis of uveitis was consistent with the Standardization Uveitis Nomenclature (SUN), and uveitis was classified as anterior, intermediate, posterior uveitis and panuveitis [10]; (ii) intraocular inflammation had been controlled and without using topical or oral corticosteroids for at least 3 months before surgery; (iii) the LogMAR BCVA of preoperation was not better than 0.5; (iv) patients were followed up for at least 3 months.

Exclusion criteria were: (i) infectious uveitis; (ii) previous intraocular surgery; (iii) patients with history of glaucoma or high myopia, optic neuropathy, media opacity other than cataract; (iv)patients with diabetes mellitus; (v) intraoperative complications like posterior capsular rupture, vitreous loss, etc.; (vi) patients younger than 18 years old or with juvenile rheumatoid arthritis; (vii) retinal or choroidal disease that could affect retinal thickness; (viii) current presence of cystoid macular edema (CME).

### Propensity score matching (PSM)

PSM analysis is a superior and more refined statistical method of adjusting for potential baseline confounding variables. To reduce bias from confounding factors between groups, a propensity score-matched analysis was done using a multivariable logistic regression model with a tolerance of 0.05. Matching covariates consisted of age, gender, type of uveitis, duration of active inflammation, unilateral or bilateral uveitis and quiescent time of ocular inflammation.

### Preoperative and postoperative examinations

An internist evaluated each patient to eliminate any significant systemic illness and confirm that the patient’s systemic disorder was in a stable period. Preoperative and postoperative examinations included BCVA (decimal visual acuity chart), intraocular pressure (IOP) measurement with the Nidek noncontact tonometer NT-4000 (Nidek Corporation., Aichi, Japan), anterior and posterior segment examinations using slit lamp biomicroscopy, central macular thickness (CMT) measured by Stratus OCT 3000 (Carl Zeiss Meditec, Inc., Dublin, CA), and corneal endothelial cell density generated by the specular microscope SP-3000P (Topcon Corporation, Tokyo, Japan). B-scan ultrasonography was performed to exclude any posterior segment pathology detectable before surgery.

Anterior chamber inflammation was measured in terms of grades of anterior chamber cells and grades of aqueous flare measured on slit lamp examination, with a 1.0 mm high and 1.0 mm wide slit beam at a 45 to 60 degree angle (Table 1) [10].

**Table 1:** Grades of anterior chamber cells and aqueous flare

Grades of anterior chamber cells		Grades of aqueous flare	
Grade	Cells in field	Grade	Description
0	< 1	0	none
+ 0.5	1-5	+ 1	faint
+ 1	6-15	+ 2	moderate (iris detail clear)
+ 2	16-25	+ 3	marked (iris detail hazy)
+ 3	26-50	+ 4	intense (fibrous exudates)
+ 4	> 50		

### Surgical management and postoperative care

Written informed consent was taken from every patient before the surgery. All surgeries were performed by same experienced consultant surgeon using topical anaesthesia. Side port incision was created using 15° disposable knife and primary main port was made with a 3.0 mm disposable keratome. Posterior synechiae and small pupils were managed by synechiolysis. In cases of dense cataract, trypan blue dye (0.06%) was used to stain anterior capsule. A 5-5.5 mm continuous circular capsulorrhexis was created after which hydrodissection, phacoemulsification and foldable hydrophobic acrylic IOL implantation in the capsular bag were performed. The patients in the IVTA group received intravitreal 3.0 mg (0.075 ml) TA injection through the pars plana 3.5 mm away from the limbus inferotemporally. After surgery they were advised to remain in a semi reclining position for 24 h.

Postoperatively, patients were detained for observation for a couple of days and appointed routinely at postoperative 1 week, 2 weeks, 1 month, 2 months and 3 months, or come to visit at any time if necessary. Additional corticosteroid (oral or invasive) was administered to control the severe postoperative inflammation in time, and patients will receive prompt and appropriate treatment in case of CME, PCO and other complications. All patients were prescribed topical levofloxacin eye drops 6 times a day for a month, prednisolone acetate 1% eye drops every 2 h daily for 1 week and compound tropicamide eye drops twice a day for 1 week. After 1 week, the frequency of the prednisolone acetate was subsequently tapered over 6 weeks with reduction of the postoperative inflammation.

### Statistical analysis

The data of this study was analysed using SPSS software version 24.0 and Graphpad Prism Software version 8.0. Descriptive statistics presented as mean (standard deviation). The BCVA readings were converted to logMAR values for statistical analysis. Continuous parameters were analysed with Student’s t-test, rank sum test was used for ranked data, and categorical parameters were analysed using chi-square test or Fisher’s exact test. The log-rank test compared the use time of corticosteroids between two groups. Differences were considered significant at a *p* value of < 0.05.

## Results

### Patient characteristics

Overall, a total of 140 patients were reviewed with complete clinical data, including 41 patients who underwent cataract surgery combined with IVTA injection and 99 patients who received cataract surgery alone. Two types of uveitic cataract found received intraoperative TA injection in our study, including anterior uveitis and panuveitis. The age and quiescent time of ocular inflammation between the two groups were not balanced (*t* = 1.991, 2.412; *p* = 0.048, 0.017). After PSM, 51 eyes of 41 patients were included in the IVTA group and 51 eyes of 41 patients were included in the control group. Pre-existing differences between groups were well balanced, and the preoperative characteristics of the patients were showed in Table 2. Table 3 showes etiological classification of uveitis in patients of two groups.

**Table 2:** Baseline characteristics before and after propensity score matching

Characteristics	Before matching (*n* = 140)			After matching (*n* = 82)		
	IVTA group (*n* = 41)	Control group (*n* = 99)	*P* value	IVTA group (*n* = 41)	Control group (*n* = 41)	*P* value
Age, mean(SD), y	42.17 (7.72)	45.90 (10.90)	0.048	42.17 (7.72)	42.07 (8.27)	0.956
Gender, n(%)			0.888			1.00
Male	21 (51.22)	52 (52.53)		21 (51.22)	21 (51.22)	
Female	20 (48.78)	47 (47.47)		20 (48.78)	20 (48.78)	
Location of uveitis, n(%)			0.599			0.647
Anterior	25 (60.98)	65 (65.66)		25 (60.98)	27 (65.85)	
Panuveitis	16 (39.02)	34 (34.34)		16 (39.02)	14 (34.15)	
Duration of active inflammation, mean(SD), m	7.74 (2.72)	6.77 (4.09)	0.162	7.74 (2.72)	6.99 (3.12)	0.246
Quiescent time of ocular inflammation, mean(SD), m	7.78 (2.28)	9.35 (3.89)	0.017	7.78 (2.28)	7.39 (2.36)	0.449
Cataract surgery, n(%)			0.915			1.00
Unilateral	31 (75.60)	74 (74.75)		31 (75.61)	31 (75.60)	
Bilateral	10 (24.40)	25 (25.25)		10 (24.39)	10 (24.40)

**Table 3:** Etiological classification of uveitis in patients of two groups

Etiology n(%)	IVTA group		Control group	
	Anterior uveitis (*n* = 25)	Panuveitis (*n* = 16)	Anterior uveitis (*n* = 27)	Panuveitis (*n* = 14)
Idiopathic	16 (64.00)	10 (62.50)	18 (66.67)	8 (57.14)
HLA-B27-associated uveitis	9 (36.00)	–	8 (29.63)	–
VKH	–	2 (12.50)	–	3 (21.43)
Behcet’s disease	–	2 (12.50)	–	1 (7.14)
Endogenous endophthalmitis	–	1 (6.25)	–	2 (14.29)
Sarcoidosis	–	1 (6.25)	1 (3.70)	–

### Visual acuity

**Fig. 1:**
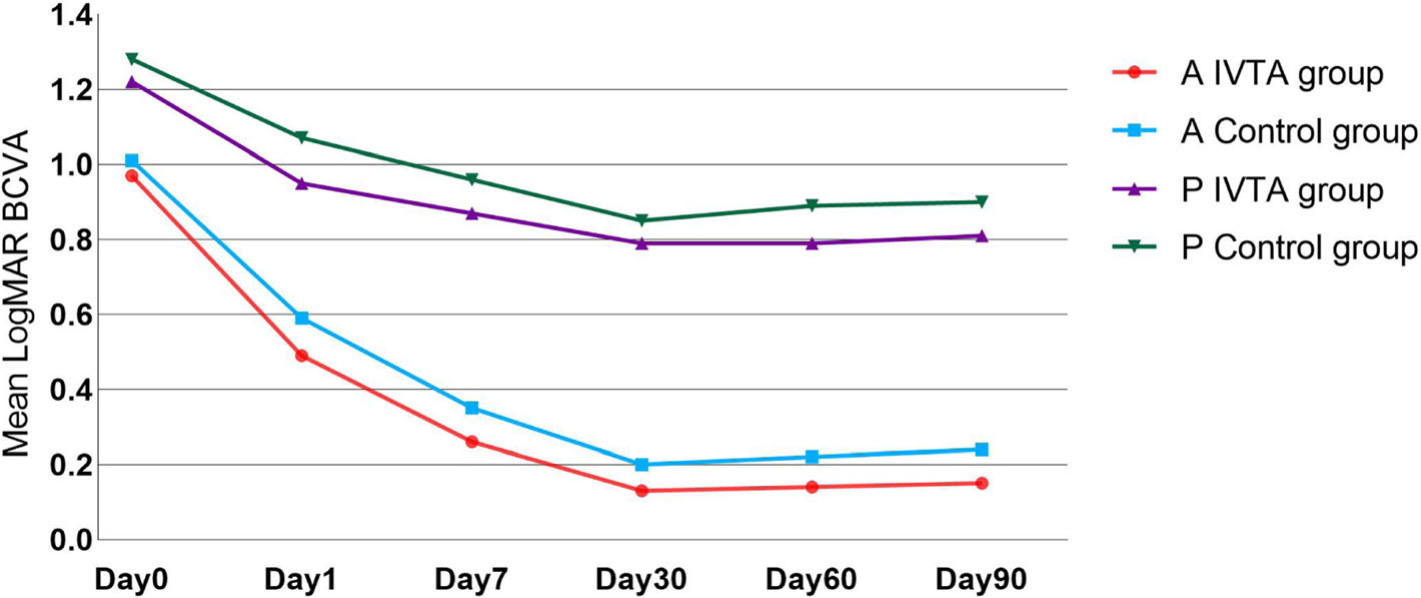
Graph showing trend of mean LogMar BCVA from preoperative to postoperative follow-up visits. (A-anterior uveitis; P-panuveitis)

Table 4 compares the BCVA in the two groups of patients at every follow-up visit. In anterior uveitis patients, the BCVA after operation in IVTA group was statistically significantly better than that in control group (*P* < 0.05). Figure 1 shows that postoperative BCVA recovered faster, and tended to be better in the IVTA group in anterior uveitis. The mean logMAR BCVA of panuveitis was better in IVTA group at postoperative follow-up visit, but there was no statistically significant difference. The main cause of no improvement in vision was pre-existing macular pathology, and recurrence of uveitis and CME were primary reasons of vision regression in late visits.

**Table 4:** Between-group comparison of LogMAR BCVA over time

	Anterior uveitis, mean(SD)		*t* value	*P* value	Panuveitis, mean(SD)		*t* value	*P* value
	IVTA group (*n* = 28)	Control group (*n* = 34)			IVTA group (*n* = 23)	Control group (*n* = 17)		
Day 0	0.97 (0.39)	1.01 (0.42)	0.376	0.709	1.22 (0.57)	1.28 (0.59)	0.288	0.775
Day 1	0.49 (0.22)	0.59 (0.21)	2.009	0.049	0.95 (0.58)	1.07 (0.56)	0.665	0.510
Day 7	0.26 (0.16)	0.35 (0.18)	2.059	0.044	0.87 (0.47)	0.96 (0.55)	0.567	0.574
Day 30	0.13 (0.13)	0.20 (0.12)	2.123	0.038	0.79 (0.47)	0.85 (0.51)	0.357	0.723
Day 60	0.14 (0.15)	0.22 (0.16)	2.015	0.048	0.79 (0.46)	0.89 (0.52)	0.664	0.511
Day 90	0.15 (0.15)	0.24 (0.15)	2.351	0.022	0.81 (0.45)	0.90 (0.53)	0.580	0.565

### Intraocular pressure (IOP)

Table 5 shows the mean IOP of two groups over time; there were no statistically significant differences at baseline and any postoperative visit (*P* > 0.05). There was increased IOP over 21 mmHg in 7 eyes, 4 out of 51 eyes (7.8%) in the IVTA group and 3 out of 51 eyes (5.9%) in the control group. One eye with ocular hypertension in the IVTA group was due to TA migrating into the anterior chamber. The transient IOP rise was managed medically with two topical anti-glaucoma eye drops for one week at most.

**Table 5:** Between-group comparison of IOP over time (*n* = 51)

	Mean IOP(SD), mmHg		*t* value	*P* value
	IVTA group	Control group		
Day 0	15.49 (3.26)	15.12 (3.40)	0.565	0.574
Day 1	15.68 (3.31)	16.14 (4.20)	0.603	0.548
Day 7	15.84 (2.96)	15.82 (2.49)	0.029	0.977
Day 14	15.86 (2.49)	15.39 (2.88)	0.882	0.380
Day 30	15.92 (2.63)	15.29 (2.85)	1.155	0.251
Day 60	15.90 (2.68)	15.33 (2.78)	1.050	0.296
Day 90	15.75 (2.62)	15.69 (3.04)	0.105	0.917

### Anterior chamber inflammation and posterior inflammation

We analysed grade of the most severe anterior chamber inflammation during postoperative 1 week hospitalisation (Table 6). The control of anterior chamber cells after operation in the IVTA group was better than that in the control group (*P <* 0.05). There was no statistically significant difference in aqueous flare between the two groups. Ten patients received bilateral sequential phacoemulsification in each group, and we found anterior chamber inflammation in the second eye was heavier or the same as in the first eye.

**Table 6:** Comparison of anterior chamber cells and aqueous flare during hospitalization (*n* = 51)

	Grade of anterior chamber cells						Grade of aqueous flare					
	Preoperative	Postoperative					Preoperative	Postoperative				
	0	0 or + 0.5	+ 1	+ 2	+ 3	+ 4	≤ + 1	0	+ 1	+ 2	+ 3	+ 4
IVTA group	51	2	27	18	4	0	51	0	1	41	9	0
Control group	51	1	20	19	8	3	51	0	1	33	13	4
*Z* value		2.119						1.871				
*P* value		0.034*						0.060*				

^*^ Fisher’s exact test

During the 3-month follow-up period, anterior uveitis recurred in 2 eyes in the IVTA group in the 5th week and 5 eyes in the control group in the 1 to 2 months. The recurrence was effectively managed with additional subconjunctival dexamethasone injections and topical corticosteroids. Panuveitis recurred in 3 eyes in the control group from 3 to 4 weeks postoperatively which was diagnosed based on clinical examination and fluorescein angiography, but none in the IVTA group. All 3 patients were given intravitreal injections of 20 mg TA, and two of them received additional oral corticosteroids. The inflammation was controlled and visual acuity was improved at the final follow-up. Of the 10 recurrent eyes, 6 eyes were the second eye of patients who underwent bilateral sequential cataract surgery, and the surgery interval was less than a month.

### Corneal endothelial cell density

Table 7 shows the corneal endothelial cell density at 1 month postoperative visit compared with the preoperative level in two groups. The postoperative density was statistically significantly lower than that preoperative in each group, while there was no significant difference between the two groups postoperatively (*p* > 0.05).

**Table 7:** Comparison of corneal endothelial cell density (*n* = 51)

	Corneal endothelial cell density, mean(SD), cell/mm^2^		*t* value	*P* value
	Day 0	Day 30		
IVTA group	2440.88 (303.54)	2146.00 (288.60)	5.028	1.00
Control group	2502.86 (281.58)	2173.55 (278.15)	5.942	1.00
*t* value	1.069	0.491		
*P* value	0.288	0.625		

### Central macular thickness(CMT)

The values of mean CMT from preoperatively to postoperatively are shown in Table 8. The OCT results of 5 eyes in the IVTA group and 6 eyes in the control group were not available because of thick opacity of cataract, so they were not included in the statistics. At postoperative 1 month, CMT was statistically lower in the IVTA group as compared to the control group. At 3 months, CMT was lower in the IVTA group than the control group, but it was not statistically significant. This might be attributed to the remission of macular edema in patients who received appropriate treatment.

**Table 8:** Between-group comparison of central macular thickness (CMT)

	CMT, mean (SD), μm		
	preoperative	postoperative	
		1 month	3 months
IVTA group(*n* = 46)	237.25 (48.36)	230.52 (45.73)	228.62 (44.30)
Control group (*n* = 45)	245.15 (53.18)	253.67 (58.45)	247.76 (52.05)
*t* value	0.614	2.107	1.890
*P* value	0.541	0.038	0.062

### Duration of continuous use of corticosteroids

**Fig. 2:**
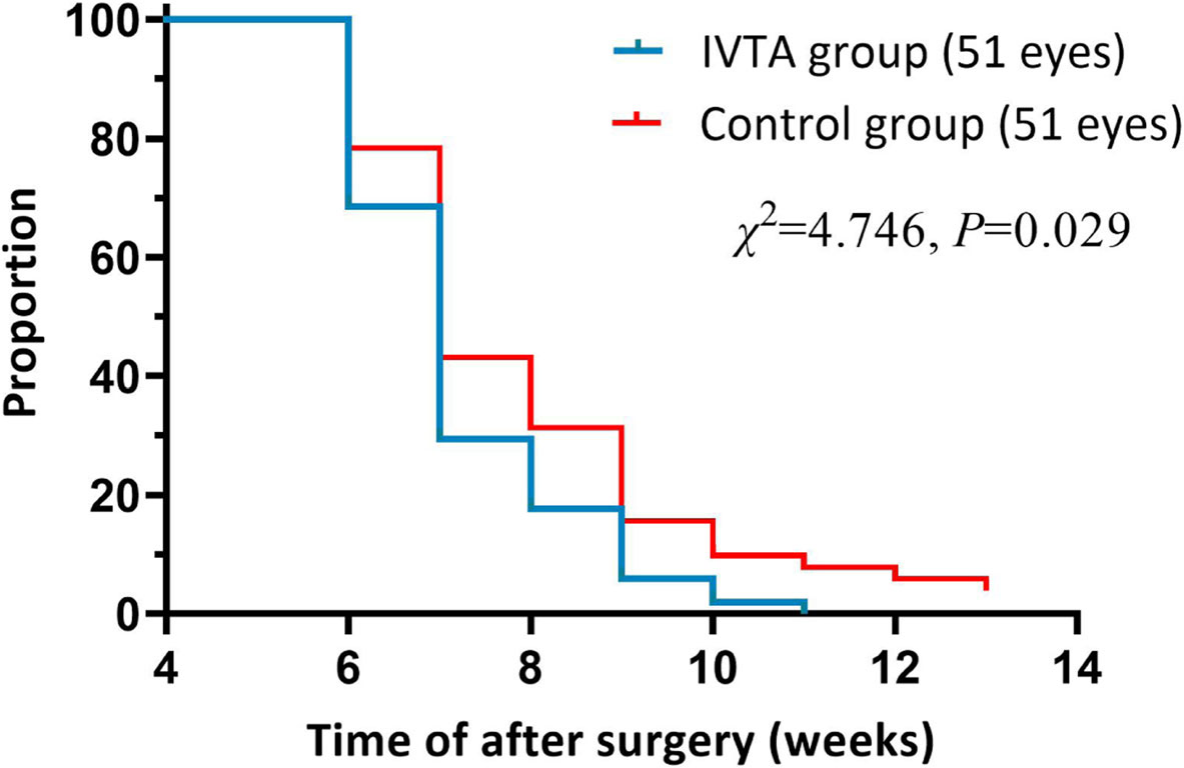
Kaplan-Meier curve showing proportion of postoperative corticosteroids use over the follow-up period in two groups

Log-rank (Mantel-Cox) analysis revealed that postoperative time of using topical or oral corticosteroids in the IVTA group is statistically reduced compared to the control group (*P* < 0.05). Figure 2 shows that in the IVTA group, the inflammation of all patients was controlled within 11 weeks, while in the control group, it took longer to taper corticosteroids to control the persistent intraocular inflammation or complications. Long-term of typical and systemic corticosteroids, or in combination with IVTA injection, were used for patients with persistent inflammation or CME in the control group.

### Complications

In the control group, a total of 7 eyes developed CME, out of which 6 eyes (13.3%) were without preoperative CME and one eye was failed to obtain the macular structure as a result of mature cataract. Most patients developed CME at around 1 month, which were treated with either oral corticosteroids or posterior sub-Tenon’s injection of 20 mg TA, depending upon severity. No patient in the IVTA group developed CME during follow-up visits.

Iris synechia mainly occurred in patients with severe postoperative anterior chamber inflammation or patients already with posterior synechiae before surgery. A total of 33 eyes developed posterior synechiae postoperatively, including 22 of 51 eyes (43.1%) in the control group and 11 of 51 eyes (21.6%) in the IVTA group.

Posterior capsular opacification requiring Nd:YAG capsulotomy developed in 8 of 51 eyes (15.7%) in the IVTA group and in 13 of 51 eyes (25.5%) in the control group.

Hyphaemia occurred in 4 of 51 eyes (7.8%) in the IVTA group and 6 of 51 eyes (11.8%) in the control group on postoperative day 1, which were resolved spontaneously in about 4 days and 7 days respectively.

## Discussion

Although there is some literature reporting the efficacy of IVTA in the treatment of uveitic cataract [5, 11], there have been no previous controlled trial comparisons. We conducted a retrospective cohort study to evaluate visual outcomes and postoperative complications after phacoemulsification combined with IVTA injection for uveitic cataract in the real world.

The lower age limit of 18 years was adopted to exclude juvenile rheumatoid arthritis, because IOL implantation in these cases may lead to more severe postoperative inflammation than other patients, in addition comprehensive treatment was applied into the postoperative management. We reviewed characteristics of 99 patients of uveitis complicated with cataract who underwent cataract surgery alone, and the mean age of which was older, and quiescent time of ocular inflammation was longer than that of IVTA group. PSM has been increasingly used as a statistical tool in observational studies. Clinical and demographic patient characteristics can be balanced between groups [12]. Potential confounding factors of this study, including age, gender, type of uveitis, duration of active inflammation, unilateral or bilateral sequential surgery and quiescent time of ocular inflammation were well-adjusted to mimic randomised controlled trial design. Patients in the IVTA group was well-matched after PSM, and the potential component of senile cataract was reduced.

In order to compare the visual outcome between two groups without regard to fundus disease, BCVA was analysed in anterior uveitis patients and panuveitis patients respectively. The BCVA improved significantly after phacoemulsification in the anterior uveitis patients, with 89.3% of patients in the IVTA group achieving 0.3 LogMAR or better visual acuity at 3 months. These results are comparable to those in previous studies showing visual acuity outcome better than 0.3 LogMAR in 67-82% cases [1, 11, 13–15]. In panuveitis patients, postoperative BCVA mildly improved in two groups, and sequelae of chronic posterior inflammation was found to be the most important factor for determining the improvement of visual acuity. The trend of postoperative BCVA improvement indicated that the application of TA resulted in early recovery in patients, which can also be appreciated on Kaplan-Meier curve. As tapering and withdrawal of corticosteroids were individually prescribed according to following examinations, short time of corticosteroids administration indicated rapid recovery. The intravitreal injection of TA inhibited the potential complications and rebound of inflammation during the postoperative visits, so patients in the IVTA group recovered quickly in the IVTA group as compared to the control group.

Preoperative control of uveitis for at least 3 months is a prerequisite to performing cataract surgery in patients with uveitis [16]. Prompt and appropriate treatment is also necessary to reverse visual deterioration in case of postoperative inflammation. According to the data, some patients had an outbreak in anterior chamber inflammation in the immediate postoperative period, especially more severe inflammation in the control group, which was progressively controlled by timely multiple subconjunctival dexamethasone injection. All 3 eyes of recurrent panuveitis and 7 eyes developed CME occurred in the control group, which inflammation and visual acuity was improved at the final follow-up, due to intravitreal or posterior sub-Tenon’s injection of TA and oral corticosteroids. Patients with panuveitis in the control group received comparable visual acuity to the IVTA group within postoperative 3 months. Regrettably, remedies brought additional pain and risk of adverse effects to patient, and postoperative inflammation lead to more patients suffering from iris posterior synechia and PCO in the control group. In addition, we found that intraoperative injection of TA seemed more necessary for these patients with refractory uveitis, including bilateral uveitis and panuveitis, which tend to severe postoperative reactions and recurrence with a high probability of oral corticosteroids application.

In our study, ocular hypertension occurred in 3 eyes (7.8%) in the IVTA group range from 22 to 30 mmHg, including one case caused by anterior chamber migration of triamcinolone crystals. The maximum of increased IOP eyes recorded in the control group was 40 mmHg, and of all 4 eyes with ocular hypertension occurred during postoperative 1 week, which could be attributed to the severe anterior chamber inflammation. There wasn’t an increased incidence of ocular hypertension, as it was supposed to be about 20.9-43.3% caused by TA injection alone reported by previous studies without cataract surgery [17–19]. Tanuj et al. showed intravitreal injection of 4 mg TA had anti-inflammatory efficacy clinically equivalent to oral corticosteroids postoperatively in uveitis patients with cataract surgery [11]. However, ocular hypertension was seen in up to 25% of patients in the IVTA group. One of the possible reasons is that a dose of 3 mg injection of TA in our study reduced the incidence of elevated IOP. Another is that 3 months quiescent period without corticosteroids use reduced the possibility of steroid-induced ocular hypertension.

Three regional TA injections have been reported to be efficient to control ocular inflammation: periocular TA, intravitreal TA and intracameral TA. Jennifer E Thorne et.al suggested that intravitreal TA was superior to periocular TA for treating uveitic macular edema with modest increases in the risk of IOP elevation [20]. Compared with intracameral injection of TA, intravitreal injection can reduce the increase of IOP and damage of corneal endothelial cell caused by high drug concentration of anterior chamber, as we found that IVTA did not aggravate the decrease of corneal endothelial cell density after phacoemulsification. In addition, combined cataract surgery would be advantageous to maintain anterior aqueous humour drug concentration through aqueous humour circulation to control anterior chamber inflammation. What’s more, IVTA could inhibit postoperative macular edema and posterior uveitis directly. Thus, IVTA is a promising approach to control anterior and posterior uveitis in cataract surgery.

Previous studies reported that triamcinolone is detectable in anterior chamber and vitreous after a single intravitreal injection for 3 to 8 months, even though triamcinolone crystals were no longer detected in the vitreous cavity on ophthalmoscopic examination [21, 22]. This suggests that one may be able to obtain relatively long-standing concentrations of triamcinolone in the eye with a single injection [21]. Our study proved that 3 mg IVTA was effective to inhibit perioperative inflammation and preserve visual acuity for at least 3 months without IOP elevation in patients received phacoemulsification. Dexamethasone implant has been introduced into clinical treatment of non-infectious uveitis with treatment benefit sustained for 6 months [23]. In a small sample size study by Gaurav et al., intraoperative intravitreal dexamethasone implant was efficacious in controlling the postoperative inflammation and reducing chances of CME without IOP elevation in uveitic cataract for 6 months [24]. But another investigation by Jennifer E Thorne et al. showed that intravitreal dexamethasone did not have a lower risk of IOP elevation than IVTA [20]. Thus, IVTA injection was not challenged its position in prevention of postcataract intraocular inflammation in patients with quiescent uveitis, and economic advantage can also be taken into account, especially in relatively underdeveloped regions. Dang et al. reported that both IVTA and dexamethasone implant could equally restore visual function and recover morphological change effectively in diabetic CME for at least 6 months, but repeated intravitreal injection was required in the IVTA group [25]. For uveitc patients with pre-existing persistent CME who have to perform cataract surgery, repeated TA intravitreal injection might be required after surgery, while dexamethasone implant could be a better choice due to its slightly long-lasting effect than TA [26]. A cost-effectiveness study comparing intravitreal dexamethasone implants versus IVTA would be necessary to provide the reference criterion for individual treatment in uveitic patients.

Cystoid macular edema is considered as the most common complication following cataract surgery in cases of uveitic cataract. According to our data, postoperative CME was seen in 6 of 45 eyes (13.3%,) control eyes, excluded one eye of CME without preoperative OCT result. The reported incidence varies and has been described to occur in the range from 2% by Suresh et al. to 33% by Estafanous et al. [4, 27, 28], which is comparable with our results. In the IVTA group, no patient developed CME in the postoperative 3 months, so to forestall inflammation is a key in avoiding severe inflammation after cataract surgery and consequently, the development of CME. Postoperative anti-inflammation afterward is not sufficient to prevent development of CME unless prophylactic oral corticosteroids were used [11].

Iris posterior synechiae is common in nearly all types of uveitic cataracts. It necessitates additional operative procedures during the surgery, which stimulate the iris and increase the risk of postoperative vascular leakage [14]. Postoperatively, the IVTA group seemed associated with a lower frequency of hyphaemia, and it spontaneously resolved faster than the control group. Previous study reported that intraoperative injection of TA at the end of diabetic vitrectomy was a useful adjunctive therapy for reducing early postoperative vitreous haemorrhage [29]. We are inspired that whether intraocular injection of TA play a role in exerting haemostasis, and further big-sample studies are necessary to better define this advantage.

Limitation of our study was that we didn’t have enough uveitic cataract patients with 3 mg IVTA injection, and data on other different types of uveitis are not available. Further large-scale and prospective randomized controlled trials comparing with other dose or new corticosteroids are necessary to highlight efficacy of 3 mg IVTA for cataract patients with some certain aetiology uveitis.

## Conclusions

Our study clearly shows that 3 mg TA intravitreal injection at the end of phacoemulsification can result in low-grade postoperative inflammation and fast vision rehabilitation, less incidence of complications (CME, iris synechia and PCO), and negligible adverse effects for 3 months, sparing the usage time of corticosteroids. So, we conclude that low-dose of 3 mg TA intravitreal injection is an effective, very safe and economic option for preventing postoperative inflammation and complications in the treatment of patients with anterior uveitis and panuveitis undergoing phacoemulsification.

## Data Availability

The analytical data in this study could be obtained from the corresponding author upon reasonable request.

## Abbreviations

TA: Triamcinolone acetonide
IVTA: Intravitreal triamcinolone acetonide
BCVA: Best corrected visual acuity
PSM: Propensity score matching
IOP: Intraocular pressure
CMT: Central macular thickness
PCO: posterior capsule opacification
CME: Cystoid macular edema
IOL: Intraocular lens
